# Design, synthesis and biological evaluation of novel hydroxy-phenyl-1H-benzimidazoles as radical scavengers and UV-protective agents

**DOI:** 10.1080/14756366.2016.1265523

**Published:** 2017-01-23

**Authors:** Alessia Bino, Anna Baldisserotto, Emanuela Scalambra, Valeria Dissette, Daniela Ester Vedaldi, Alessia Salvador, Elisa Durini, Stefano Manfredini, Silvia Vertuani

**Affiliations:** a Department of Life Sciences and Biotechnology, School of Pharmacy and Health Products, University of Ferrara, Master Course in Cosmetic Sciences and Technology, Laboratory of Health Products, Ferrara, Italy;; b Department of Pharmaceutical and Pharmacological Sciences, University of Padua, Padua, Italy

**Keywords:** Benzimidazole, antioxidant, UV booster, reactive oxygen species, sunscreens

## Abstract

An ever-increasing incidence of skin neoplastic diseases is registered. Therefore, it is important to protect the skin from the UV radiation that reaches the epidermis and dermis but also to block ROS generated by them. Our attention was attracted in developing new compounds provided with both UV filtering and antioxidant capacities. To this end, 2-phenyl-1H-benzimidazole-5-sulfonic acid (PBSA), a known UV filter, was selected as lead compound for its lack of antioxidant activity, high water solubility and good safety profile. PBSA was sequentially modified introducing hydroxyls on the phenyl ring and also substituting the functional group in position 5 of the benzimidazole ring. At the end of the synthetic study, a new, very potent class of antioxidants has been obtained. Surprisingly some of the developed molecules, while devoid of significant UV-filtering activity was endowed with potent UV-filtering booster capability if associated with known commercial UVB and UVA filters.

## Introduction

Human exposure to UV radiation causes different acute and chronic effects on the skin; acute responses include photodamage, erythema, synthesis of vitamin D, tanning and most dangerous immunosuppression and mutation, which are responsible of chronic UVR (ultraviolet radiation) effects such as photocarcinogenesis^1^. UVA and UVB are a small portion of the total radiation that reaches the earth, but they cause damage to eyes, hair and they are responsible for inducting various skin disorders including skin cancer outcomes^2^^,^^3^, because they have a high content of energy. UVB rays are responsible for direct damage, such as sunburn, while UVA rays induce indirect damage caused, in most cases, to the formation of oxidizing species^4^.

The skin is the largest organ of the body, covering the entire surface of the body and is continuous with the mucous membranes; it is the most vulnerable organ and affected by UV rays. In addition to being the organ most exposed to solar radiation, the skin is rich in chromophores, molecules able to absorb the radiation energy, such as melanin, DNA, RNA, proteins, lipids, transurocanic acid, and aromatic amino acids, as tyrosine and tryptophan^5^. In the past, UVA radiation has been much undervalued and was considered not dangerous, unlike UVB radiation has always been considered the most dangerous for tissues. Currently, it is known that UVA rays have a greater ability to penetrate the skin, they manage to reach the dermis and are responsible for consequential damages due to generation of reactive oxygen species (ROS). ROS are highly reactive species that include superoxide anion radical, hydroxyl radical, singlet oxygen (^1^O_2_), and hydrogen peroxide (H_2_O_2_), each of which can further trigger the generation of ROS^6^^,^^7^. When the skin is exposed to UV radiation for long periods without adequate precautions, the body is no longer able to neutralize the radical species generated, triggering the mechanisms of photoaging, immunosuppression, and photocarcinogenesis^3^^,^^8^. Oxidative stress causes premature aging of cells and tissues that become more permeable and lose their efficiency. The damage to the skin generated by ROS, however, can also extend to the loss of the barrier function of the stratum corneum, the promotion of inflammatory processes, erythema, up to the cancer^8^^,^^9^. Both UVB and UVA radiation contribute to photoaging and UV-generated ROS seem responsible for mitochondrial DNA mutations and protein oxidative modifications. Collagen is particularly affected by oxidation and degradation that is carried out by matrix metalloproteases; the synthesis of these enzymes increases following signaling pathways initiated by reactive oxygen species. Furthermore, large collagen degradation products inhibit new collagen synthesis and so, collagen degradation itself negatively regulates new collagen synthesis and then interstitial collagen is reduced and damaged.

Photoaging, involves (i) elastosis, that clinically is characterized by yellowish discoloration of the skin, and (ii) rough surface, for an excessive growth of elastic fibers degraded and greater amount of basic substance, composed mostly of glycosaminoglycans and proteoglycans. In addition, keratinocytes, in photoaged epidermis, can be irregular with a loss of polarity, a condition known as actinic keratosis clinically perceived as red, rough, hyperkeratotic patches; actinic keratosis has been demonstrated as the initial injury that may progress to invasive squamous cell carcinoma^9–11^. The UV filters used in the pharmaceutical and/or cosmetic fields can either form, in general, a protective layer on the skin surface and themselves absorb the solar radiation, thus preventing that the same penetrate into the deeper layers of the skin. Once the filtering molecule has absorbed the energy of the solar radiation, one of the methods to disperse this energy is the transformation or degradation of the molecule itself, which thus becomes photounstable^12^. This change can trigger the formation of oxidizing radical species. Therefore, in addition to the loss of filtering capacity, one has to consider also cell damages resulting from the formation of these ROS^13^. Since detrimental effects of UV rays are also triggered by over-production of reactive oxygen species, it is important to protect the skin reducing the UV radiation that reaches epidermis and dermis but also trying to scavenge the reactive oxygen species.

Our research group is involved, since several years, in the study of antioxidant molecules^14^, so we focused our attention on the possible synthesis of dualistic molecules able to act, at the same time, as UV protectants and inhibitors of the formation of radical species. According to this purpose, the initial phase of the study regarded screening of commercial molecules, and provided, as one of the best candidate, 2-phenyl-1H-benzimidazole-5-sulfonic acid (PBSA), chosen as a lead compound for the lack of antioxidant activity, high solubility in water and good safety profile. PBSA, commonly used in commercial sunscreens, provides good protection against UVB rays, although it lacks filtering capacity regarding UVA radiation and, moreover, appears to induce the production of reactive species of oxygen after irradiation, with potentially harmful effects^15^^,^^16^. With the purpose to achieve antioxidant activity, maintaining the filtering capacity, PBSA was modified introducing hydroxyl groups on the phenyl ring and also substituting the functional group in position 5 of the benzimidazole ring, to evaluate the influence of this moiety on filtering and antioxidant capabilities. To assess the antioxidant power of the obtained molecules, *in vitro* complementary tests were performed (DPPH, PCL and FRAP assays), then we incorporated the new molecules in a standard topical formulation to evaluate the UV-filtering capacity *in vitro* and also to verify the antioxidant power of the finished formulations. The new molecules have also submitted to toxicity and phototoxicity studies to exclude adverse effects of the new products. It is fundamental for photoprotection that a filtering molecule remains active during exposure to sunlight: similar UV filters, available on market, unfortunately suffer of evident photoinstability. After this screening, only the molecules that showed both good filtering activity and antioxidant capacity, and that were devoid of cytotoxicity and phototoxicity, were considered, at the end of the first phase of the study, for photostability: formulations containing the selected compounds were exposed to solar simulated radiation and then tested by accelerated stability studies.

### Experimental section

#### General procedures

Reactants, solvents and standard samples were purchased from Sigma-Aldrich, Milan, Italy. Reaction course was routinely monitored by thin-layer chromatography on pre-coated silica gel plates (Macherey-Nagel Durasil-25) by detection under a 254-nm UV lamp and/or by spraying the plates with 1% FeCl_3_ solution in water and using as eluent dichloromethane/methanol (90:10) or butanol/water/acetic acid (60:20:20).

The molecular weights of the compounds were determined by ESI (Micromass ZMD 2000), and the values are expressed as [MH]^+^. ^1^H-NMR spectra were determined in d*6*-DMSO and recorded on VXR-200 Varian spectrometer and Mercury Plus-400. Chemical shifts are expressed in parts per million (δ) relative to the TMS internal standard. UV spectrophotometric analyses were carried out on a UV-VIS spectrophotometer (ThermoSpectronic Helios γ, Cambridge, UK). HPLC analysis was performed using an Agilent 1100 Series HPLC System equipped with a G1315A DAD, autosampler and with a Phenomenex Synergi Hydro-RP C18 80 Å column (4.6 × 150 mm, 4 μm). Photostability studies were carried out with a solar simulator device (Suntest CPS+; Atlas, Linsengericht, Germany) equipped with a Xenon lamp, an optical filter to cut off wavelengths shorter than 290 nm and an IR-block filter to avoid thermal effects.

#### Materials

3,4-Diamino-benzensulfonic acid (**1**) has been prepared according to known procedures^17^.

#### Chemistry synthesis

##### 2-(4-Hydroxy-phenyl)-1H-benzimidazole-5-sulfonic acid (2)

In a round-bottomed flask (50 mL) equipped with a magnetic stirrer, to a solution of 3,4-diamino-benzene sulfonic acid (**1**) (100 mg, 0.35 mmol) in ethanol (5 mL) was added a solution of sodium bisulfite 1N in water (0.7 mL, 0.7 mmol) and 4-hydroxy-benzaldehyde (46 mg, 0.35 mmol); the reaction mixture was heated at 80 °C under reflux for 24 h. The solvent was then evaporated under reduced pressure, the solid was washed with HCl 1N to precipitate the interest product, the suspension was filtered and the solid washed with methanol to afford **2** (60 mg, 0.21 mmol, yield 60%) as a light yellow powder. ^1^H NMR (400 MHz, [D_6_]DMSO): δ = 13.4–15.8 (*s*, broad, 2H, –SO_3_H, –NH), 10,78 (*s*, broad, 1H, –OH), 8.06 (d, 2H, aryl, *J*= 8.8 Hz), 7.78 (d, 1H, benzimidazole, *J*= 8.4 Hz), 7.73 (d, 1H, benzimidazole, *J*= 8.4 Hz),7.91 (*s*, 1H, benzimidazole), 7.09 ppm (d, 2H, aryl, *J*= 9.2 Hz). ESI–MS: *m*/*z* calculated for C_13_H_10_N_2_O_4_S + H^+ ^[M + H^+^]: 290.30. Found: 290.50.

##### 2-(3,4-Dihydroxy-phenyl)-1H-benzimidazole-5-sulfonic acid (3)

In a round-bottomed flask (50 mL) equipped with a magnetic stirrer, to a solution of 3,4-diamino-benzene sulfonic acid (**1**) (100 mg, 0.35 mmol) in ethanol (5 mL) was added a solution of sodium bisulfite 1N in water (0.7 mL, 0.7 mmol) and 3,4-dihydroxy-benzaldehyde (48 mg, 0.35 mmol); the reaction mixture was heated at 80 °C under reflux for 24 h. The solvent was then evaporated under reduced pressure, the solid was washed with HCl 1N to precipitate the interest product, the suspension was filtered and the solid washed with methanol to afford **3** (60 mg, 0.2 mmol, yield 57%) as a white powder. ^1^H NMR (400 MHz, [D_6_]DMSO): δ= 13.8–15.6 (*s*, broad, 2H, –SO_3_H, –NH), 10.4 (*s*, broad, 1H, –OH), 9.75 (*s*, broad, 1H, –OH), 7.88 (*s*, 1H, benzimidazole), 7.75 (dd, 1H, benzimidazole, *J*_ortho_= 8.4 Hz, *J*_meta_= 1.6 Hz), 7.68 (d, 1H, benzimidazole, *J*= 8.4 Hz), 7.52–7.56 (*m*, 2H, aryl), 7.05 ppm (d, 1H, aryl, *J*= 8 Hz). ESI–MS: *m*/*z* calculated for C_13_H_10_N_2_O_5_S + H^+ ^[M + H^+^]: 306.29. Found: 306,7.

##### 2-(2-Hydroxy-phenyl)-1H-benzimidazole-5-sulfonic acid (4)

In a round-bottomed flask (50 mL) equipped with a magnetic stirrer, to a solution of 3,4-diamino-benzene sulfonic acid (**1**) (100 mg, 0.35 mmol) in ethanol (5 mL) was added a solution of sodium bisulfite 1N in water (0.7 mL, 0.7 mmol) and 2-hydroxy-benzaldehyde (40 μL, 0.35 mmol); the reaction mixture was heated at 80 °C under reflux for 24 h. The solvent was then evaporated under reduced pressure, the solid was washed with HCl 1N to precipitate the interest product, the suspension was filtered and the solid washed with methanol to afford **4** (94 mg, 0.32 mmol, yield 91%) as a white powder. ^1^H NMR (400 MHz, [D_6_]DMSO): δ= 11.9–14.2 (*s*, broad, 3H, –OH, –SO_3_H, –NH), 8.06 (dd, 1H, *J*_ortho _= 8 Hz, *J*_meta_= 2.4 Hz), 8.04 (*s*, 1H, benzimidazole), 7.75–7.80 (*m*, 2H), 7.56–7.60 (*m*, 1H, aryl), 7.20 (d, 1H, aryl, *J*= 7.6 Hz), 7.13–7.17 ppm (*m*, 1H, phenyl). ESI–MS: *m*/*z* calculated for C_13_H_10_N_2_O_4_S + H^+ ^[M + H^+^]: 290.30. Found: 290.8.

##### 2-(2,4-Dihydroxy-phenyl)-1H-benzimidazole-5-sulfonic acid (5)

In a round-bottomed flask (50 mL) equipped with a magnetic stirrer, to a solution of 3,4-diamino-benzene sulfonic acid (**1**) (100 mg, 0.35 mmol) in ethanol (5 mL) was added a solution of sodium bisulfite 1N in water (0.7 mL, 0.7 mmol) and 2,4-dihydroxy-benzaldehyde (48 mg, 0.35 mmol); the reaction mixture was heated at 80 °C under reflux for 24 h. The solvent was then evaporated under reduced pressure, the solid was washed with HCl 1N to precipitate the interest product, the suspension was filtered and the solid washed with methanol to afford **5** (65 mg, 0.21 mmol, yield 61%) as a white powder. ^1^H NMR (400 MHz, [D_6_]DMSO): δ= 13.87 (*s*, broad, 2H, –SO_3_H, –NH), 11.74 (*s*, broad, 1H, –OH), 10.66 (*s*, broad, 1H, –OH), 7.99 (*s*, 1H, benzimidazole), 7.90 (d, 1H aryl, *J*_ortho _= 8.8 Hz), 7.69–7.75 (*m*, 2H, benzimidazole), 6.611 (*s*, 1H, aryl), 6.57 ppm (dd, 1H, aryl, *J*_ortho _= 8.8 Hz, *J*_meta_= 2.4 Hz). ESI–MS: *m*/*z* calculated for C_13_H_10_N_2_O_5_S + H^+ ^[M + H^+^]: 306.29. Found: 306.7.

##### 2-(2,3,4-Trihydroxy-phenyl)-1H-benzimidazole-5-sulfonic acid (6)

In a round-bottomed flask (50 mL) equipped with a magnetic stirrer, to a solution of 3,4-diamino-benzene sulfonic acid (**1**) (100 mg, 0.35 mmol) in ethanol (5 mL) was added a solution of sodium bisulfite 1N in water (0.7 mL, 0.7 mmol) and 2,3,4-trihydroxy-benzaldehyde (54 mg, 0.35 mmol); the reaction mixture was heated at 80 °C under reflux for 24 h. The solvent was then evaporated under reduced pressure, the solid was washed with HCl 1N to precipitate the interest product, the suspension was filtered and the solid washed with methanol to afford **6** (82 mg, 0.26 mmol, yield 73%) as a whitish powder. ^1^H NMR (400 MHz, [D_6_]DMSO): δ= 13.8–14 (*s*, broad, 3H, –SO_3_H, –NH, –OH), 10.45 (*s*, broad, 1H, –OH), 9.2 (*s*, broad, 1H, –OH), 7.99 (*s*, 1H, benzimidazole), 7.70–7.78 (*m*, 2H, benzimidazole), 7.42 (d, 1H, aryl, *J*_ortho_ = 8.8 Hz), 6.64 ppm (d, 1H, aryl, *J*_ortho_ = 8.8 Hz). ESI–MS: *m*/*z* calculated for C_13_H_10_N_2_O_6_S + H^+ ^[M + H^+^]: 322.29. Found: 322.4.

##### 2-(4-Hydroxy-phenyl)-1H-benzimidazole (7)^18^

The data are in agreement with those reported in literature.

##### 2-(3,4-Dihydroxy-phenyl)-1H-benzimidazole (8)^19^

The data are in agreement with those reported in literature.

##### 2-(2,4-Dihydroxy-phenyl)-1H-benzimidazole (9)^18^

The data are in agreement with those reported in literature.

##### 2-(2-Hydroxy-phenyl)-1H-benzimidazole (10)^18^

The data are in agreement with those reported in literature.

##### 2-(2,3,4-Trihydroxy-phenyl)-1H-benzimidazole (11)

In a round-bottomed flask (50 mL) equipped with a magnetic stirrer, to a solution of o-phenylenediamine (100 mg, 0.92 mmol) in ethanol (5 mL) was added a solution of sodium bisulfite 1N in water (1.84 mL, 1.84 mmol) and 2,3,4-trihydroxy-benzaldehyde (142 mg, 0.92 mmol); the reaction mixture was heated at 80 °C under reflux for 24 h. The solvent was then evaporated under reduced pressure, the solid was washed with HCl 1N to precipitate the interest product, the suspension was filtered and the solid washed with methanol to afford **11** (208 mg, 0.86 mmol, yield 94%) as a light brown powder. ^1^H NMR (400 MHz, [D_6_]DMSO): δ= 14 (*s*, broad, 2H, –NH, –OH), 10.4 (*s*, broad, 1H, –OH), 9.2 (*s*, broad, 1H, –OH), 7.77–7.782 (*m*, 2H, benzimidazole), 7.59 (d, 1H, aryl, *J*_ortho_ = 8.8 Hz), 7.45–7.50 (*m*, 2H, benzimidazole), 6.66 ppm (d, 1H, aryl, *J*_ortho_= 8.8 Hz). ESI–MS: *m*/*z* calculated for C_13_H_10_N_2_O_3_ + H^+ ^[M + H^+^]: 242.23. Found: 242.6.

##### 2-(3,4-Dihydroxy-phenyl)-1H-benzimidazole-5-carboxylic acid (12)^20^

In a round-bottomed flask (50 mL) equipped with a magnetic stirrer, to a solution of 3,4-Diaminobenzoic acid (100 mg, 0.66 mmol) in ethanol (5 mL) was added a solution of sodium bisulfite 1N in water (1.32 mL, 1.32 mmol) and 3,4-dihydroxy-benzaldehyde (91 mg, 0.66 mmol); the reaction mixture was heated at 80 °C under reflux for 24 h. The solvent was then evaporated under reduced pressure, the solid was washed with HCl 1N to precipitate the interest product, the suspension was filtered and the solid washed with methanol to afford **12** (135 mg, 0.5 mmol, yield 76%) as a light brown powder. ^1^H NMR (400 MHz, [D_6_]DMSO): δ= 10.5 (*s*, broad, 1H, –OH), 9.8 (*s*, broad, 1H, –OH), 8.25 (*s*, 1H), 8.05 (dd, 1H, *J*_ortho_= 8.4 Hz, *J*_meta_= 1.6 Hz), 7.82 (d, 1H, *J*= 8.4 Hz), 7.69–7.73 (*m*, 2H), 7.06 ppm (d, 1H, *J*= 8 Hz). ESI–MS: *m*/*z* calculated for C_14_H_10_N_2_O_4_ + H^+ ^[M + H^+^]: 270.24. Found: 270.7.

##### 2-(2,4-Dihydroxy-phenyl)-1H-benzimidazole-5-carboxylic acid (13)^21^

The data are in agreement with those reported in literature.

##### 2-(4-Hydroxy-phenyl)-1H-benzimidazole-5-carboxylic acid (14)^22^

The data are in agreement with those reported in literature.

##### 2-(2,3,4-Trihydroxy-phenyl)-1H-benzimidazole-5-carboxylic acid (15)

In a round-bottomed flask (50 mL) equipped with a magnetic stirrer, to a solution of 3,4-diaminobenzoic acid (100 mg, 0.66 mmol) in ethanol (5 mL) was added a solution of sodium bisulfite 1N in water (1.32 mL, 1.32 mmol) and 2,3,4-trihydroxy-benzaldehyde (102 mg, 0.66 mmol); the reaction mixture was heated at 80 °C under reflux for 24 h. The solvent was then evaporated under reduced pressure, the solid was washed with HCl 1N to precipitate the interest product, the suspension was filtered and the solid washed with methanol to afford **15** (151 mg, 0.53 mmol, yield 80%) as a light pink powder. ^1^H NMR (400 MHz, [D_6_]DMSO): δ= 13–14 (*s*, broad, 3H, –OH –COOH, –NH), 10.2–11 (*s*, broad, 2H, –OH), 8.26 (d, 1H, *J*_meta_= 1.6 Hz), 8.02 (dd, 1H, *J*_ortho_= 8.6 Hz, *J*_meta_= 1.6 Hz), 7.82 (d, 1H, *J*_ortho_= 8.6 Hz), 7.58 (d, 1H, *J*_ortho_= 8.8 Hz), 6.65 ppm (d, 2H, *J*_ortho_= 8.8 Hz). ESI–MS: *m*/*z* calculated for C_14_H_10_N_2_O_5_ + H^+ ^[M + H^+^]: 286.24. Found: 286.4.

#### Antioxidant activity assays

##### Photochemiluminescence (PCL) method

PCL assay, based on the methodology of Popov and Lewin^23^, was used to measure the antioxidant activity of synthesized compounds with a Photochem® apparatus (Analytik Jena, Leipzig, Germany) against superoxide anion radicals generated from luminol, a photosensitizer, when exposed to UV light (Double Bore® phosphor lamp, output 351 nm, 3 mWatt/cm^2^). The antioxidant activity was measured using ACL (Antioxidant Capacity of Liposoluble substance) kit provided by the manufacturer designed to measure the antioxidant activity of lipophilic compounds^24^. In ACL studies, the kinetic light emission curve, which exhibits no lag phase, was monitored for 180 s and expressed as micromoles of Trolox per gram of compound. The areas under the curves were calculated using the PCLsoft control and analysis software. As greater concentrations of Trolox working solutions were added to the assay medium, a marked reduction in the magnitude of the PCL signal and hence the area calculated from the integral was observed. This inhibition was used as a parameter for quantification and related to the decrease in the integral of PCL intensities caused by varying concentrations of Trolox. The observed inhibition of the signal was plotted against the concentration of Trolox added to the assay medium. The concentration of the added tested compounds was such that the generated luminescence during the 180-s sampling interval fell within the limits of the standard curve. The antioxidant assay was carried out in triplicate for each sample, and 20 μL of the diluted compound in HPLC-grade methanol (ACL) was sufficient to correspond to the standard curve.

##### 1,1-Diphenyl-2-picryl-hydrazyl radical (DPPH) assay

To 1.5 mL DPPH methanolic solution (0.5 mM) was added 0.750 mL of sample solution proper diluted. Samples absorbance measurements were evaluated with a UV-VIS spectrophotometer (Thermo Spectronic Helios γ, Cambridge, UK) at fixed wavelength of 517 nm. Blank sample was prepared adding methanol to DPPH solution and Trolox was used as standard reference to achieve a calibration curve. The radical-scavenging activity is expressed as inhibition ratio of initial concentration of DPPH radical and is calculated according to the formula: Inhibition percentage (*I*_p_) = [(AB-As)/AB]·100; where AB and A_s_ are, respectively, the absorbance values of blank reaction and of the tested sample^25^.

##### Ferric reducing antioxidant of potency (FRAP) assay

The ferric reducing ability of each standard solution was measured according to a modified protocol described by Guihua et al.^26^. The reagent for analysis was freshly prepared by mixing the following solutions in the reported ratio 10/1/1 (v:v:v) (i) 0.1 M acetate buffer pH 3.6, (ii) TPTZ (2,4,6-Tri(2-pyridyl)-s-triazine) 10 mmol/L in 40 mmol/HCl, (iii) ferric chloride 20 mmol/L. To a 1.9 mL of reagent were added 0.1 mL of sample proper diluted or solvent when blank was performed. Readings at fixed wavelength of the absorption maximum (593 nm) were done after 30 min, using a UV-VIS spectrophotometer; it was evaluated the absorbance increase of sample solution against the absorbance of blank reaction as parameter to calculate the antioxidant activity. The antioxidant activity is given as Trolox activity since this standard was used to perform the calibration curves.

#### Cosmetic formulations

The synthesized molecules were included at the concentration of 3% in a topical formulation, to prove the effective filtering capacity and the antioxidant activity of finished formulation. It was decided to use a standard formulation oil in water (O/W).

INCI: Aqua, glycerin, phenoxyethanol, methylparaben, ethylparaben, butylparaben, propylparaben, isobutylparaben, cetyl alcohol, glyceryl stearate, PEG-75 stearate, ceteth-20, steareth-20, cetyl alcohol, dimethicone, C12–15 alkyl benzoate, cocoglycerides, NaOH solution 10%.

##### Evaluation of filtering parameters

*In vitro* approaches consist in applying a thin film of sunscreen product on an artificial substrate and test, via spectrophotometric measures, the amount of UV radiation passing through the film. Several different artificial substrates are available for this type of analysis; the substrate should be as closed as possible to the physical characteristics of the skin. Among the substrates available for these purposes, the substrate used in this study to analyse the sunscreen products were PMMA plates. This substrate is easily handled and can be supplied with a reproducible roughness. WW5 PMMA plates have been purchased from Schonberg GmbH (Munich, Germany). The plates, used in this study, have an area of 25 cm^2^ and standardized 5 m roughness. Transmittance and absorbance measurements were carried out by a SHIMAZDU UV-2600 provided of integrating sphere ISR 2600 60 mm and coupled with a SPF determination software and a PMMA plate with approximately 15 μL of glycerin served as reference.

#### Cytotoxicity and phototoxicity tests

##### Cellular culture

An immortalized, nontumorigenic cell line of human keratinocytes (NCTC-2544) was grown in a DMEM medium (Sigma-Aldrich Milan, Italy), supplemented with 115 units/mL of penicillin G, 115 μg/mL streptomycin, and 10% fetal calf serum (Invitrogen, Milan, Italy). Individual wells of a 96-well tissue culture microtiter plates (Falcon, Becton–Dickinson) were inoculated with 100 μL of complete medium containing 5 × 10^3^ NCTC-2544. The plates were incubated at 37 °C in a humidified (5% CO_2_) incubator for 18 h prior to the experiments.

##### Cytotoxicity

After medium removal, 100 μL of the drug solution, dissolved in DMSO and diluted in DMEM medium, was added to each well, incubated at 37 °C for 72 h. Final DMSO concentration never exceeded 0.5%. Cell viability was assayed by the MTT (3–(4,5-dimethylthiazol-2-yl)-2,5 diphenyl tetrazolium bromide) test as previously described by Mosmann^27^.

##### Irradiation procedure

Two HPW 125 Philips lamps, mainly emitting at 365 nm, were used for UVA irradiation experiments. The spectral irradiance of the source was 4.0 mWcm^−^^2^ as measured at the sample level by a Cole-Parmer Instrument Company radiometer (Niles, IL) equipped with a 365-CX sensor. One or two PL-S 9 W/12 Philips lamps (280–370 nm; peak at 315 nm) were used for UVB irradiation experiments. To restrict the incident radiation to the range 305–370 nm, a glass filter (Schott SWG-305) was used. Total energy was detected by the same equipped with a sensor (model CX-312).

##### Cellular photoprotection experiments

After medium removal, 100 μL of the drug solution, dissolved in DMSO and diluted with Hank’s Balanced Salt Solution (HBSS pH =7.2), was added to each well, incubated at 37 °C for 30 min, and then irradiated (20 J/cm^2^ for UVA and 0.5 and 1 J/cm^2^ for UVB). After irradiation, the solution was replaced with the medium and the plates were further incubated for 48 h. Cell viability was assayed by the MTT [(3–(4,5-dimethylthiazol-2-yl)-2,5 diphenyl tetrazolium bromide)] test as previously described by Mosmann^27^.

#### HPLC analysis

HPLC analysis was performed using an Agilent 1100 Series HPLC System equipped with a G1315A DAD, and with a Hydro-RP C18 Synergi 80 Å column (4.6 × 150 mm, 4 μm) from Phenomenex, maintained at 25 °C during all the time of the analysis. The mobile phase consisted of water (0.01 M H_3_PO_4_) (solvent A) and acetonitrile (0.01 M H_3_PO_4_) (solvent B).

1. **PBSA** and **3**: the determination was carried out in isocratic condition, A: 91%/B: 9%. Separation was monitored with absorbance detection at λ_max_ of the molecule ±8 nm. The flow rate was 1.0 mL/min.
2. **10**: the determination was carried out in isocratic condition, A: 84%/B: 16%. Separation was monitored with absorbance detection at λ_max_ of the molecule ±8 nm. The flow rate was 1.0 mL/min

The sample solutions were filtered by a 0.45-μm filter, before be injected into column (HPLC filters were purchased from Chemtek Analitica, Bologna, Italy).

#### Photostability studies

The solar simulator emission was maintained at 500 W/m^2^. A portion of cosmetic formulation containing the sunscreen molecule (3%, w/w) was transferred onto the bottom of a beaker to gain a cosmetic mount of 2 mg/cm^2^ and then was irradiated for 1 h with the solar simulator. After irradiation the beaker was removed and its content quantitatively transferred into a 50 mL calibrated flask with methanol and the remaining sunscreen concentration was determined by HPLC as described above. All samples were protected from light both before and after irradiation, the degree of photodegradation was evaluated by comparing the areas of the irradiated samples, with those of the unirradiated preparations.

## Statistical evaluations

Relative standard deviations and statistical significance (Student’s *t-*test; *p* ≤ 0.05) were given where appropriate for all data collected. One-way analysis of variance (ANOVA) and LSD *post hoc* Tukey’s honest significant difference test were used for comparing the bioactive effects of different samples. All computations were made using the statistical software STATISTICA 6.0 (StatSoft Italia srl, Vigonza, Italy).

## Results and discussion

### Chemistry

**Scheme 1.**
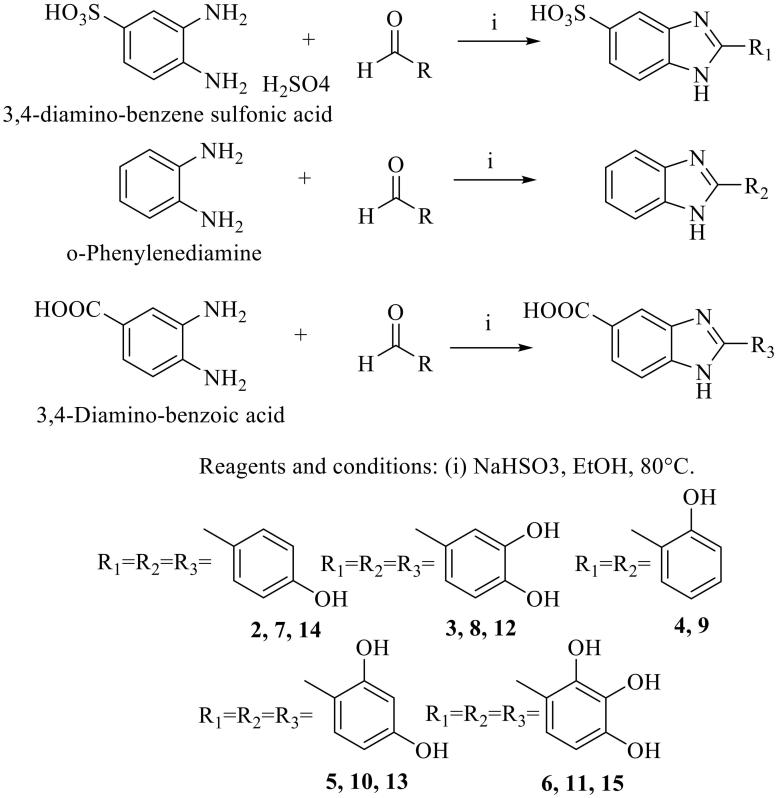
Synthesis of compounds **2–15**.

Three groups of molecules were synthesized: the first group maintained the sulfonic acid moiety in position 5 of the benzimidazole ring (**2–6**); the second group showed no functional group on the benzimidazole ring (**7–11**), and the third group presented, instead of the sulfonic acid, the carboxylic acid moiety (**12–15**) (Scheme 1). For the first group of molecules, the synthesis of the benzoimidazole was accomplished from 3,4-diamino-benzene sulfonic acid (**1**) that was in turn obtained from o-phenylenediamine and 96% sulfuric acid^17^. 5(6)-Substituted benzimidazoles are a well-known class of heterocycles, which displays tautomerism at the imidazole ring^28^^,^^29^. Different synthetic procedures described in the literature were analyzed to obtain the desired products. The best solution was the use of sodium bisulfite as a catalyst; all reactions were carried out in ethanol at reflux for 24 h^30^. The same procedure was used to synthesize 2-(4-hydroxy-phenyl)-1H-benzimidazole-5-sulfonic acid (**2**), 2-(3,4-dihydroxy-phenyl)-1H-benzimidazole-5-sulfonic acid (**3**), 2-(2-hydroxy-phenyl)-1H-benzimidazole-5-sulfonic acid (**4**), 2-(2,4-dihydroxy-phenyl)-1H-benzimidazole-5-sulfonic acid (**5**), 2-(2,3,4-trihydroxy-phenyl)-1H-benzimidazole-5-sulfonic acid (**6**), 2-(4-hydroxy-phenyl)-1H-benzimidazole (**7**), 2-(3,4-dihydroxy-phenyl)-1H-benzimidazole (**8**), 2-(2,4-dihydroxy-phenyl)-1H-benzimidazole (**9**), 2-(2-hydroxy-phenyl)-1H-benzimidazole (**10**), 2-(2,3,4-trihydroxy-phenyl)-1H-benzimidazole (**11**), 2-(3,4-dihydroxy-phenyl)-1H-benzimidazole-5-carboxylic acid (**12**), 2-(2,4-dihydroxy-phenyl)-1H-benzimidazole-5-carboxylic acid (**13**), 2-(4-hydroxy-phenyl)-1H-benzimidazole-5-carboxylic acid (**14**), 2-(2,3,4-trihydroxy-phenyl)-1H-benzimidazole-5-carboxylic acid (**15**).

### Antioxidant activity assays

All the synthesized compounds were tested to determine their antioxidant capacity by PCL (photochemoluminescence) analysis, DPPH and FRAP tests (Table 1). The lead PBSA, as expected, was devoided of any activity in both DPPH and PCL assays, showing only minimal activity in FRAP assay. Compounds with only one phenolic hydroxyl, in position 2 (**5** and **10**) or 4 (**2**, **7**, **14**) on the phenyl ring, have shown the lowest antioxidant capacity in all performed assays. The antioxidant activity increased adding another hydroxyl group; products with two hydroxyls in position 2 and 4 on the phenyl moiety (**5**, **9** and **13**) showed improved activity, but the best results were obtained with **3**, **8**, **12** with hydroxyl moieties in position 3 and 4 of the phenyl ring. The gap, in terms of antioxidant power, between the different positional isomers was very high in all performed tests. Compounds with three hydroxyls on phenyl ring (**6**, **11** and **15**) did not show any further improvement in antioxidant activity as compared to the parent bis-hydroxyls in position 3 and 4 of the phenyl ring. Interestingly, the results for DPPH test were even lower than those of **3**, **8** and **12** and also significant decrease in antioxidant activity was shown in PCL assay. In FRAP analysis, compounds with three hydroxyl moieties gave results comparable to that of molecules with hydroxyls in position 3 and 4. Considering the three assays, molecules with better antioxidant profile were those with two phenolic hydroxyls in position 3 and 4; in all performed tests, compound **3** showed the highest antioxidant capacity, followed by compound **8** and **12**. For compounds **6**, **11** and **15,** with three hydroxyls, and compounds **5**, **9** and **13,** with two hydroxyls in position 2 and 4 on the phenyl ring, the antioxidant capacity decreased probably because of hydrogen bond formation between nitrogen of benzimidazole and the hydroxyl group in position 2 on the phenyl ring.

**Table 1.** Results of antioxidant assays. Each value was obtained from three experiments (Mean ± SE).

Product	DPPH (μmolTrolox/mmol)(*p* ≤ 0.05)	FRAP (μmolTrolox/mmol)(*p* ≤ 0.05)	PCL (μmolTrolox/mmol)(*p* ≤ 0.05)
PBSA	< LOQ^a^	0.79 ± 0.06	< LOQ^a^
**2**	2.04 ± 0.15	12.23 ± 0.25	11.36 ± 0.08
**3**	2145.31 ± 45.8	2707.29 ± 29.52	22,838 ± 836,26
**4**	2.03 ± 0.09	16.22 ± 0.89	2.06 ± 0.05
**5**	21.41 ± 043	19.87 ± 0.22	159.1 ± 4.3
**6**	1362.12 ± 133.96	2713.3 ± 45.17	1924.35 ± 101.82
**7**	1.67 ± 0.08	21.94 ± 0.12	9.66 ± 0,04
**8**	1974.58 ± 16.89	2663.21 ± 32.53	19190.6 ± 443.18
**9**	16.47 ± 0.54	37.3 ± 0.35	198.53 ± 2.65
**10**	0.87 ± 0.05	23.04 ± 0.09	10.93 ± 0.05
**11**	1811.02 ± 61.7	2723.19 ± 35.74	1614.675 ± 19.95
**12**	1771.82 ± 84.75	2433.28 ± 21.74	13174.31 ± 240.68
**13**	32.48 ± 2.38	31.63 ± 1.96	160.26 ± 4.09
**14**	1.185 ± 0.02	2.62 ± 0.06	6.98 ± 0.27
**15**	1241.03 ± 9.43	2355.15 ± 52.55	1515.65 ± 75.56

LOQ^a^ limit of quantification.

### Antioxidant activity assays

**Figure 1.**
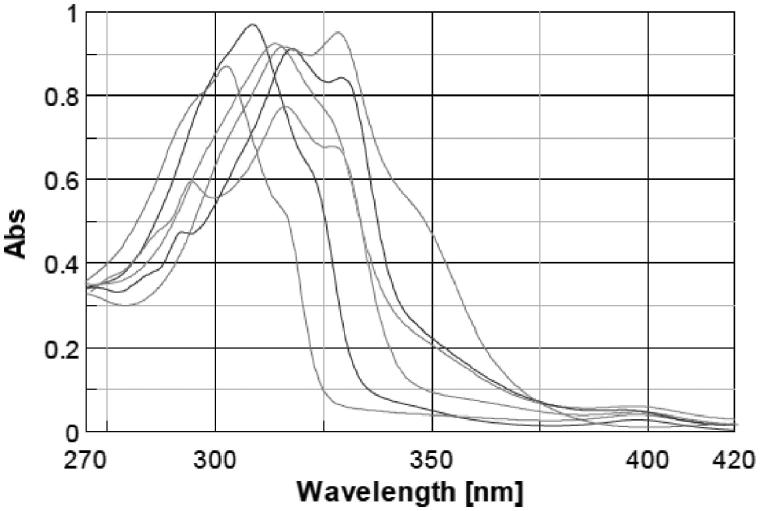
Comparison between spectra of PBSA and molecules with sulfonic acid moiety on benzimidazole ring: **2**, **3**, **4**, **5**, **6**.

**Figure 2.**
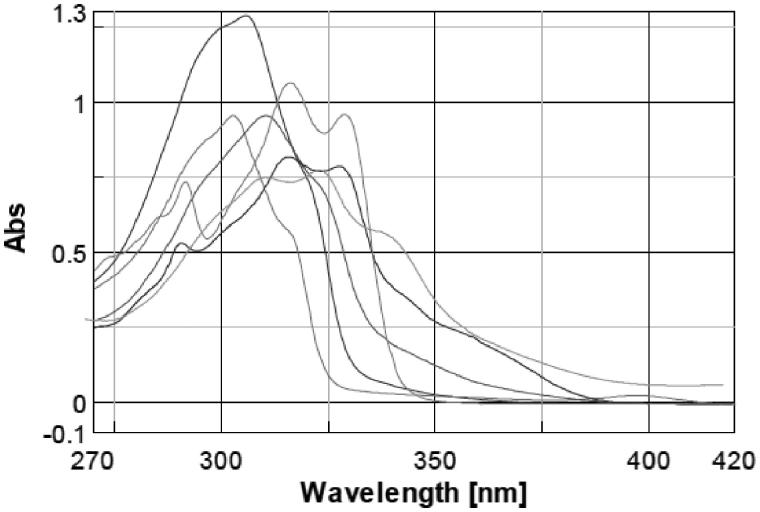
Comparison between spectra of PBSA and molecules without substituents on benzimidazole ring: **7**, **8**, **9**, **10**, **11**.

**Figure 3.**
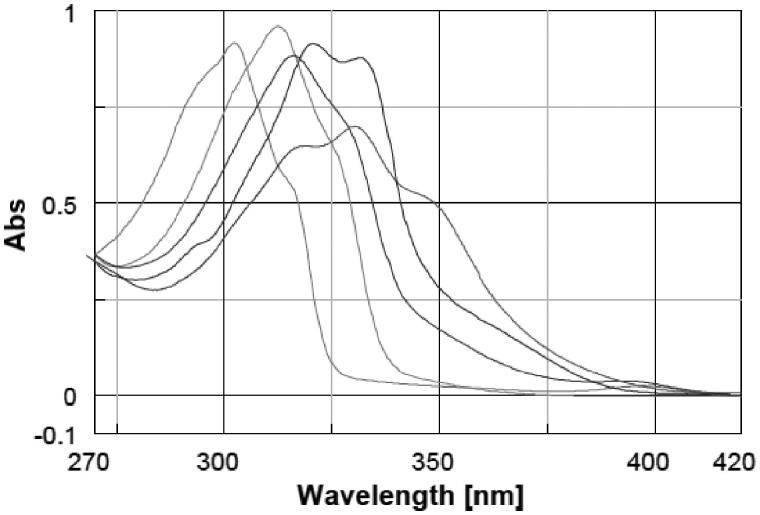
Comparison between spectra of PBSA and molecules with carboxylic acid moiety on benzimidazole ring: **12**, **13**, **14**, **15**.

Sun protection factor (SPF) is related to the UV absorption of the substances, so before including the molecules in cosmetic formulations we evaluated the wavelength of maximum absorption (λ_max_) and the molar extinction coefficient (ɛ) of each molecule in aqueous solution at pH 7. The UV spectra were recorded between 270 and 420 nm (20 nm higher and lower then UVA and UVB range) to verify the spectrum profile of the molecules within UVA and UVB region. UV spectrum of PBSA is an UVB filter and presents a λ_max_ of 302 nm; after this peak, the absorbance fades going to higher wavelength until almost zero around 325 nm, and then, it provides no absorption in the UVA region. Molecules spectra overlapped for category according to the substituent in position 5 of the benzimidazole ring are shown in Figures 1, 2 and 3; PBSA spectrum is presented in all figures for comparison.

**Figure 4.**
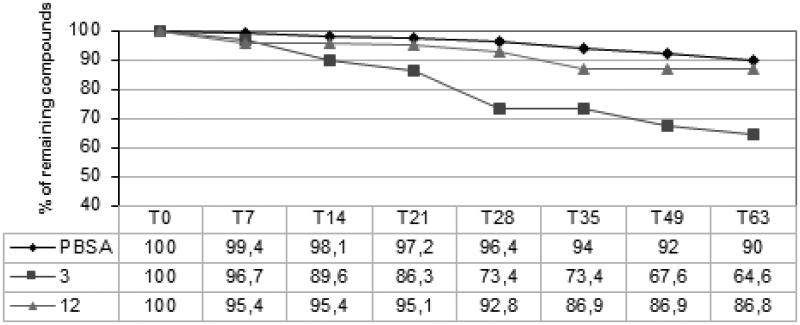
Stability studies in formulation (formulations were submitted to accelerated aging at 40 °C for 63 days).

From the spectra appears that the lambda maxima (Table 2) of the new molecules shifted toward higher wavelength and in general the range of absorption curve was wider than PBSA. Within a group of molecules, the batochromic shift was more marked for the molecules that have a hydroxyl moiety in position 2 of the phenyl ring (**4**, **5**, **6**, **9**, **10**, **11**, **13** and **29**); the effect of ortho substituted is known to produce this shift toward higher wavelength^31^. Increasing the number of auxochrome groups on phenyl ring an increment in the absorption range occurred, compounds with three hydroxyl on the phenyl ring (**6**, **11**, **15**) had the broadest spectrum and their lambda maxima shifted at the highest wavelength compared to the other compounds. The absorption spectrum of molecules with the same number and position of hydroxyl seemed only slightly influenced by the substituent in position 5 of the benzimidazole ring. After having established the lambda maxima for every compound, solutions at different concentrations were prepared to calculate the molar extinction coefficient by means of linear regression and applying Lambert–Beer equation (Table 2).

**Table 2.** Values of λ max and ɛ.

	λ max (nm)	ɛ
**PBSA**	302	25 000
**2**	308	25 000
**3**	313	23 000
**4**	315	21 000
**5**	318	23 000
**6**	328	22 200
**7**	306	24 000
**8**	311	16 000
**9**	315	14 000
**10**	315	17 000
**11**	325	12 000
**12**	317	21 000
**13**	321	20 000
**14**	312	24 000
**15**	332	17 000

### Topical formulation evaluation

In order to evaluate possible activity as sun filters and antioxidants, suitable in the development of novel class of dualistic sunscreens, the synthesized molecules were included at the concentration of 3% (w/w) in a topical formulation. To this end standard oil in water (O/W) formulation was devised, taking into account the absence of any UV filtering and/or antioxidant molecule, in order to obtain a reference formulation. Data obtained are shown in Table 4. It was not possible to analyze compound 7 in the formulation because of high instability (phase separation induced by 7). Formulations containing the active ingredients were tested for their SPF efficacy by the *in vitro* method derived from Diffey and Robson^32^, in comparison with the same formulation containing the reference PBSA.

This method is based on the measure of spectral transmission of ultraviolet radiation, with and without the sunscreen applied, through an irregular substrate that simulates the skin surface and permits transmission of UV radiation. Although with some limitation, the test is used to predict SPF *in vivo* and to screen the filtering activity of new molecules that cannot be tested by *in vivo* because of lacking of more data concerning safety. The critical wavelength (λ_c_) for the test product is defined as that wavelength where the area under the absorbance spectrum for the irradiated product from 290 nm to λ_c_ is 90% of the integral of the absorbance spectrum from 290 nm to 400 nm. Critical wavelength value of greater than 370 nm is necessary, in order to satisfy requirements for broad-spectrum UVB/UVA protection and associated labeling. Polymethylmethacrylate plates (PMMA) with a specific roughness were a satisfactory substrate for this method, because they are UV-transparent, nonfluorescent, photostable and inert to all potential sunscreen formulation ingredients. Table 3 lists the data obtained for the model formulation containing the synthesized compounds. These parameters are necessary to evaluate whether a sunscreen fulfills the requirements for efficacy, and they are SPF, UVA protection factor (UVAPF), UVA/UVB ratio and critical wavelength. The standard formulation was also analyzed by this method and did not exhibit any protecting activity.

**Table 3:** . Protecting efficacy^a^.

Formulatedcompounds	SPF (*p* ≤ 0.05)	UVA/UVB (*p* ≤ 0.05)	UVAPF (*p* ≤ 0.05)	λc^b^(nm)
**PBSA**	4.56 ± 1.09	0.26 ± 0.05	1.04 ± 0.06	333
**2**	4.74 ± 0.15	0.51 ± 0.01	1.36 ± 0.07	346
**3**	9.83 ± 0.64	0.74 ± 0.04	3.63 ± 0.23	358
**4**	2.22 ± 0.13	0.96 ± 0.01	1.06 ± 0.07	355
**5**	2.18 ± 0.14	1.13 ± 0.01	1.21 ± 0.04	370
**6**	5.99 ± 0.12	0.89 ± 0.03	2.72 ± 0.08	383
**8**	5.20 ± 0.20	0.78 ± 0.02	1.90 ± 0.09	368
**9**	3.57 ± 0.23	1.10 ± 0.05	1.89 ± 0.05	380
**10**	1.96 ± 0.14	1.01 ± 0.02	1.06 ± 0.08	378
**11**	2.85 ± 0.22	0.90 ± 0.01	1.35 ± 0.1	381
**12**	2.21 ± 0.17	1.00 ± 0.04	1.88 ± 0.12	370
**13**	2.85 ± 0.17	1.27 ± 0.02	1.58 ± 0.10	368
**14**	6.01 ± 0.31	0.70 ± 0.05	2.04 ± 0.11	350
**15**	3.69 ± 0.24	0.96 ± 0.08	1.87 ± 0.08	382

a Values are the mean of three independent determinations, each of which at least five readings.

b Wavelength at which the integral of the spectral absorbance curve reaches 90% of the area under the curve from 290 to 400 nm.

**Table 4.** Antioxidant activity of finished formulations^a^.

Formulation	PCL μmol Trolox/g formulation(*p* ≤ 0.05)
**PBSA**	<LOQ^b^
**2**	0.33 ± 0.03
**3**	2212.09 ± 68.35
**4**	0.06 ± 0.02
**5**	0.95 ± 0.02
**6**	101.98 ± 1.08
**8**	2972.107 ± 145.63
**9**	8.66 ± 0.04
**10**	0.5 ± 0.02
**11**	153.35 ± 8.14
**12**	1373.39 ± 19.43
**13**	4.97 ± 0.17
**14**	0.16 ± 0.02
**15**	86.99 ± 6.95

a Values are the mean of three independent determinations.

b Limit of quantification.

Data obtained showed that all the new molecules have improved filtering parameters UVA, since UVA protection factor, UVA/UVB ratio and critical wavelength are higher than those of the lead compound. In particular, molecules with phenolic hydroxyl in the ortho position on the phenyl ring (**4**, **5**, **9**, **10** and **13**) had better and higher UVA/UVB ratio and critical wavelength, although they have the SPF value less than lead compound. Formulation containing products **2** and **14,** which have a phenolic hydroxyl at the para position on the phenyl ring, have shown good values in terms of SPF, greater UVA protection factor, critical wavelength and also higher UVA/UVB ratio than PBSA, but yet lower than the molecules containing hydroxyl on the phenyl ring in ortho. Compounds **3**, **8** and **12**, with the hydroxyl in position 3 and 4, were really interesting for their high levels of SPF: **3** showed a SPF twice as PBSA. This product shows also great UVA protection factor, UVA/UVB ratio and critical wavelength than PBSA. Compound **8** has lower SPF than **3**, but higher than PBSA; in comparison with **3**, molecules **8** and **12** presented higher UVA/UVB ratio and critical wavelength. Regarding compounds with three hydroxyls on phenyl ring, they have good UVA filtering parameters, their critical wavelengths are the highest and SPF is higher than PBSA for molecule **6**, while SPF of **15** is slightly lower and decreases further in molecule **11**. Thus, it can be observed that parameters are mainly influenced by the number and position of the hydroxyl groups on the phenyl ring rather than the substituent in the 5-position of benzimidazole. Concomitantly, all formulations were analyzed to assess whether the antioxidant power was maintained. In these regards, we have previously demonstrated that PCL is a very useful method for the determination of antioxidant capacity of skin products because applicable to both raw material and finished products^33^. In Table 4, the antioxidant activity, measured by PCL method, of finished formulations is reported; as it can be seen results were in agreement with that obtained testing the pure compounds. Formulations containing compounds with only one hydroxyl (**2**, **4**, **10** and **14**) had poor antioxidant capacity, which slightly increased for formulations having compounds with two hydroxyls in position 2 and 4 on the phenyl ring (**5**, **9** and **13**). Formulations containing compounds **3**, **8** and **12** presented highest antioxidant capabilities; these are followed, but with important decrease in antioxidant capacity, by formulations with compounds **6**, **8** and **15** that have three hydroxyl moieties on phenyl ring.

### Cytotoxicity and phototoxicity tests

Compounds used in sunscreens must be stable upon irradiation thus, to ensure the safety of the synthesized compounds, specific cytotoxicity and phototoxicity assays were performed. The tests were conducted using a specific cell line of human keratinocytes (NCTC-2544). To assess cell viability, in the presence of the study compounds, before and after the irradiation, the MTT assay was performed^27^. The cytotoxicity was checked after 72 h from the incubation of the keratinocytes with compounds; moreover, the same experiments were performed in the presence of the parent PBSA. Table 5 displays cytotoxicity test results, expressed in IC_50_ (μM), which is the concentration required to inhibit 50% of cellular growth.

**Table 5.** Cytotoxicity results expressed as IC_50_ values on NCTC-2544 cells.

Compounds	IC_50_ (μM)
**PBSA**	>50
**2**	26.4 ± 2.7
**3**	>50
**4**	>50
**5**	>50
**6**	23.1 ± 2.9
**7**	26.1 ± 2.4
**8**	>50
**9**	>50
**10**	>50
**11**	>50
**12**	>50
**13**	>50
**14**	>50
**15**	>50

Most of the compounds showed no cytotoxicity at the concentration used in the cell line of human keratinocytes. Only compounds **2**, **6** and **7**, had IC_50_ values in the range between 20 and 30 μM, in particular compound **6** showed the lowest IC_50_ and then was the most cytotoxic among the tested compounds. Molecules were verified for their photocytotoxicity in the same cell line; the cells were treated with 50 μM solutions of compound and after 30 min were irradiated with 20 J/cm^2^ UVA or with UVB at two energy amounts: 0.5 J/cm^2^ and 1 J/cm^2^ UVB. The compounds **2**, **6** and **7** were used at a concentration of 20 μM to exclude any antiproliferative effect of these compounds. After irradiation, the solution was replaced with growth medium and the cells were further incubated for 48 h, and then, cell viability was assessed by MTT assay. The results are presented in Table 6 as percentages of cell survival in comparison with nonirradiated cells (100% cell survival). The phototoxicity of tested molecules was compared with the ratio of cell survival of irradiated keratinocytes without substance (IC = irradiated control).

**Table 6.** Phototoxicity test data.

Compound	Concentration (μM)	% Cell survival		
		UVA 20 J/cm^2^	UVB 0.5 J/cm^2^	UVB 1 J/cm^2^
**IC**		69.1 ± 3.2%	63.1 ± 3.2%	33.8 ± 3.1%
**PBSA**	50	74.5 ± 1.4%	75.2 ± 4.5%	64.2 ± 3.1%
**2**	20	77.3 ± 2.8%	78.0 ± 3.5%	46.7 ± 3.4%
**3**	50	77.4 ± 4.0%	71.5 ± 2.5%	58.1 ± 2.6%
**4**	50	76.9 ± 2.0%	74.3 ± 3.2%	37.7 ± 1.7%
**5**	50	79.0 ± 6.0%	70.8 ± 3.9%	38.6 ± 3.7%
**6**	20	18.4 ± 2.3%	59.3 ± 3.4%	29.7 ± 2.1%
**7**	20	72.8 ± 2.9%	74.2 ± 1.3%	44.0 ± 4.1%
**8**	50	62.9 ± 4.3%	72.8 ± 1.0%	43.9 ± 3.4%
**9**	50	3.7 ± 0.5%	1.1 ± 0.3%	0.5 ± 0.1%
**10**	50	4.8 ± 0.6%	19.8 ± 2.4%	3.5 ± 0.3%
**11**	50	1.2 ± 0.2%	45.6 ± 2.6%	27.0 ± 2.4%
**12**	50	74.3 ± 2.0%	76.3 ± 3.6%	64.5 ± 3.5%
**13**	50	80.7 ± 1.3%	69.0 ± 0.2%	57.5 ± 2.4%
**14**	50	76.7 ± 3.4%	74.2 ± 3.4%	58.4 ± 2.5%
**15**	50	12.2 ± 1.3%	60.1 ± 2.3%	25.6 ± 2.7%

Differences in cell survival rate were detected; molecules **6**, **9**, **10**, **11** and **15** seemed to have a phototoxic effect as cell survival decreased considerably in comparison to irradiated control, thus these compounds were judged not suitable for further development as photoprotective agents. All the other compounds, including PBSA, increased cell survival after UVA and UVB irradiation in comparison with irradiated control. A great increase in cell viability was observed after UVB irradiation (1 J/cm^2^), in particular in presence of PBSA and of compounds **3**, **12**, **13** and **14** the cell survival doubled in relation to the control. The most interesting compounds regarding the phototoxicity test were **3**, **12**, **13** and **14** that increased cell survival both after UVA and UVB irradiation, hence these substances seemed suitable as photoprotective molecules.

### Photostability and stability studies

Stability and photostability tests were performed only on compounds that as provided with the best antioxidant capacity together with a good photoprotection activity (i.e. **3**, and **12**). At first, the effective concentration of filtering molecules was verified in the formulation by HPLC. Samples of the formulations (2 mg/cm^2^) were spread on a PMMA plate, for each compound including PBSA, and placed under a solar simulator for one hour before repeating HPLC analysis. Results reported in Table 7 showed that the two compounds have photostability comparable to the lead compound. Compound **3** was slightly less photostable than PBSA while the best result was achieved with compound **12** that exhibited the lowest degradation rate, less than 2%, and therefore had better photostability than lead compound.

**Table 7.** Evaluation of photostability^a^.

Product	Residual product (*p* ≤ 0.05)
PBSA	96.7% ± 1.8%
**3**	94.9% ± 0.8%
**12**	98.4% ± 0.9%

a Values are the mean of four independent experiments.

Molecules **3** and **12** were challenged in accelerated stability studies against the parent compound. The formulations were submitted to accelerated aging at 40 °C and analyzed at specific intervals, with HPLC.

The trend reported in Figure 4 showed that PBSA had the highest stability, followed by compound **12**, and finally compound **3**. This was a somewhat expectable result because molecules with antioxidant activity are more susceptible to degradation phenomena.

**Figure 5.**
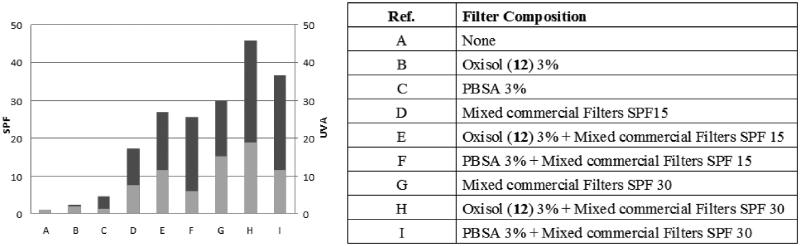
SPF values (reported in dark grey) and UVA-PF values (reported in light grey) of the tested formulations.

### SPF booster properties

Considering all the results, that is, filtering and antioxidant capacity, cytotoxicity, phototoxicity, stability and photostability, the molecule that best complied all the requirements was compound **12** (also termed oxisol)^28^. Thus, because those properties were quite interesting we decided to further investigate the sunscreen performance of this molecule, in comparison with PBSA and, as usual for sunscreens, in combination with commercial organic filters.

A set of model formulations was prepared: an oil-in-water emulsion (O/W), without sunscreen molecules (Ref. A, Figure 5), prepared as reference formulation; two formulations containing PBSA or oxisol (**12**) at 3% were prepared (Ref. C and B, respectively, Figure 5), in order to evaluate the filter properties of oxisol (**12**). Moreover, as stated earlier, SPF booster properties were assayed on other four formulations including a mix of filters with a theoretical SPF value of 15 and 30, adding 3% of oxisol (**12**) or PBSA (Ref. E, F, G, H, Figure 5). The tested formulations (Ref. D, and G, Figure 5) contain a fixed combination of commercial organic filters, butyl methoxydibenzoylmethane–octocrylene–ethylhexyl methoxycinnammate, that are dosed to perform a theoretical value of SPF 15 and SPF 30 as calculated by BASF SunScreen Simulator^34^, as reported in Table 8.

**Table 8.** Mixed commercial filters used in the formulations.

UV filter composition	SPF 15	SPF 30
INCI name	Amount	Amount
Butyl methoxydibenzoylmethane	2.3%	4.7%
Ethylhexyl methoxycinnamate	4.0%	8.0%
Octocrylene	4.0%	8.0%

Figure 5 reports the results obtained for all the emulsions in term of SPF and UVA-PF values.

As expected, the reference formulation does not present sunscreen properties. The comparison between the emulsions containing only oxisol (**12**) and PBSA highlights the capability of PBSA to behave as a “sun-filter”, while oxisol (**12**) does not present an appreciable value of SPF. Considering the performance of the two molecules in association with a mix of filters it can be observed that, as expected PBSA deals with the SPF (UVB rays) without influence on the UVA component of the spectrum. Remarkably, Oxisol (**12**) influences both the parameters.

### Preliminary in vivo evaluation of products

The *in vivo* method according to ISO 24444:2010 standard and European Recommendation 647/2006 was applied to determine the SPF value for 3 selected sunscreen products reported in Table 9, with the aim to confirm the booster effect of Oxisol (**12**). In this study, five subjects per product, female and male, 20 and 35 aged, were used for the preliminary test. Their skin phototype was chosen with sun sensitivity categories of type I, II and III according to Fitzpatrick. A skin area on the back, 35 cm^2^, was irradiated with different UV doses so that the minimal erythema dose (MED) was determined after 24 h. Prior to the *in vivo* tests, the sunscreen products were tested with the *in vitro* test method to ensure that severe deviations from the predictive SPF values were taken into account. All volunteers had been informed in details before signing a written declaration of consent. For *in vivo* SPF determination, a Multiport UV Solar Simulator Model 601, 300 W, was used. This sun simulator emits ultraviolet radiation in the region between 290 and 400 nm from six independent outputs. Each output is adjusted on scalar UV doses, set according to parameters tabulated by the instrument. All sunscreen products were applied in a thin film of 2.00 ± 0.05 mg/cm^2^ in the selected area on the back. The product distribution was reached by a gentle massage using a fingercoat, at least 15 min before the irradiation started.

**Table 9.** *In vivo* evaluation of selected formulations.

Emulsion	*In vivo* SPF
Oxisol (12) 3%	2.68
Mixed filters SPF 15	15.78
Oxisol (12) 3%+Mixed Filters SPF15	21.74

## Conclusions

In general, all new synthesized compounds have demonstrated an antioxidant efficacy greater than the lead compound (PBSA), but the best were **3**, **8** and oxisol (**12)**, which showed a good activity of filtering even with high antioxidant power (also maintained in the cosmetic formulation). Compounds **3**, **8** and oxisol (**12)** met the requirements for good filtering and antioxidant activity; they were also free of cytotoxic and phototoxic effects and, in particular, **3** and oxisol (**12)** showed a good photoprotection against UVA and UVB radiation. Photostability studies suggested that the most interesting compounds are **3,** which degrades only slightly more than PBSA, but out of all, oxisol (**12)** showed photostability superior to lead compound with a degradation rate of less than 2%. Considering all the results together, filtering and antioxidant capacity, cytotoxicity, phototoxicity, stability and photostabilty, the molecule that best complied all the requirements was oxisol (**12**). Oxisol (**12**) represents the first example of development of a booster molecule provided of very potent antioxidant activity and capable to improve activity of known sunscreen. Thus, although it cannot be defined as a sunscreen filter, it is very useful in combination with conventional UV filter to improve potency (less amount of UV filter needed) and also express potent antioxidant activity, very important to improve protection offered by traditional sunscreens also protecting against harmful oxidative species produced by solar radiation. We believe that this result opens new perspective in the development of a new class of protective molecules that consent to save up to 37% of the traditional UV filters.
